# Enhancing axonal myelination in seniors: A review exploring the potential impact cannabis has on myelination in the aged brain

**DOI:** 10.3389/fnagi.2023.1119552

**Published:** 2023-03-22

**Authors:** Colin J. Murray, Haley A. Vecchiarelli, Marie-Ève Tremblay

**Affiliations:** ^1^Neuroscience Graduate Program, University of Victoria, Victoria, BC, Canada; ^2^Division of Medical Sciences, University of Victoria, Victoria, BC, Canada; ^3^Départment de Médicine Moléculaire, Université Laval, Québec City, QC, Canada; ^4^Axe Neurosciences, Center de Recherche du CHU de Québec, Université Laval, Québec City, QC, Canada; ^5^Neurology and Neurosurgery Department, McGill University, Montréal, QC, Canada; ^6^Department of Biochemistry and Molecular Biology, University of British Columbia, Vancouver, BC, Canada; ^7^Centre for Advanced Materials and Related Technology (CAMTEC), University of Victoria, Victoria, BC, Canada; ^8^Institute for Aging and Lifelong Health, University of Victoria, Victoria, BC, Canada

**Keywords:** aged brain, myelination, myelin repair, cannabis, microglia, oligodendrocyte, oligodendrocyte progenitor cell, astrocyte

## Abstract

Consumption of cannabis is on the rise as public opinion trends toward acceptance and its consequent legalization. Specifically, the senior population is one of the demographics increasing their use of cannabis the fastest, but research aimed at understanding cannabis’ impact on the aged brain is still scarce. Aging is characterized by many brain changes that slowly alter cognitive ability. One process that is greatly impacted during aging is axonal myelination. The slow degradation and loss of myelin (i.e., demyelination) in the brain with age has been shown to associate with cognitive decline and, furthermore, is a common characteristic of numerous neurological diseases experienced in aging. It is currently not known what causes this age-dependent degradation, but it is likely due to numerous confounding factors (i.e., heightened inflammation, reduced blood flow, cellular senescence) that impact the many cells responsible for maintaining overall homeostasis and myelin integrity. Importantly, animal studies using non-human primates and rodents have also revealed demyelination with age, providing a reliable model for researchers to try and understand the cellular mechanisms at play. In rodents, cannabis was recently shown to modulate the myelination process. Furthermore, studies looking at the direct modulatory impact cannabis has on microglia, astrocytes and oligodendrocyte lineage cells hint at potential mechanisms to prevent some of the more damaging activities performed by these cells that contribute to demyelination in aging. However, research focusing on how cannabis impacts myelination in the aged brain is lacking. Therefore, this review will explore the evidence thus far accumulated to show how cannabis impacts myelination and will extrapolate what this knowledge may mean for the aged brain.

## 1. Introduction

Research looking into the potential therapeutic benefits offered by cannabis has drastically increased since its legalization (in Canada, medicinal: 2001, recreational: 2018) in many countries around the world. The proposed therapeutic benefits of cannabis consumption are numerous, ranging from pain management to a potential aid in multiple sclerosis (MS; Whiting et al., 2015; Paes-Colli et al., 2022). The possible benefit offered in MS—an autoimmune disease characterized by demyelination—introduces an interesting association between cannabinoids, the biologically active compounds found in cannabis, and myelination (Longoria et al., 2022).

Myelination is an essential process that involves the efficient and deft wrapping of myelin—a lipid rich sheath—around the axons of neurons by oligodendrocytes in the central nervous system (CNS). This wrapping facilitates rapid propagation of electrical signals and is essential for neuronal synchronization and proper communication between discrete regions of the brain. In adulthood, the total net level of myelin in the brain is relatively constant, but the myelin sheaths themselves turnover in a slow conserved cycle between degradation and regeneration, a process facilitated by oligodendrocytes (Buscham et al., 2019; Aber et al., 2022; Meschkat et al., 2022). However, this homeostatic cycle is lost in the aged brain, leading to an abnormal deposition of myelin and a net decline in myelin content (Peters, 2009; Rivera et al., 2022). This decline is evident in the healthy aged brain and is furthermore a common characteristic of many neurodegenerative diseases associated with aging (Guttmann et al., 1998; Bartzokis, 2004; Cox et al., 2016; Coelho et al., 2021; Furber et al., 2022). Importantly, this loss is tightly linked to cognitive decline (Bartzokis, 2004; Bennett and Madden, 2014; Wang et al., 2020; Coelho et al., 2021).

Due to the prevalence of demyelination in the aged brain and its association with cognitive decline, there is an urgent need to better understand and alleviate the burdens of this process. Certain lifestyle factors such as diet and exercise have recently emerged as a promising way to improve cognition and brain health throughout the lifespan. A controversial lifestyle factor that has relatively unknown cellular effects on the brain during aging is cannabis use.

In recent years, seniors (aged 65+) have increased their use of cannabis faster than any other demographic in North America, possibly as a result of some combination of destigmatization, legalization, and increased accessibility (Salas-Wright et al., 2017; Han and Palamar, 2020; Keethakumar et al., 2021). However, research focusing on cannabis use in seniors is scarce, not to mention research specific to its impact on myelination. Therefore, it is prudent to identify what impact cannabis use has on the integrity of myelin in the aged brain in order to propose harm reduction strategies if needed. Alternatively, the potential for cannabinoids to therapeutically target demyelinating diseases points to an ability for cannabis to regulate the myelination process in a beneficial way. This lack of evidence regarding the beneficial or detrimental outcomes of cannabis on myelination in the aged brain highlights a clear gap in the literature. By excluding seniors, the currently available research is omitting a large portion of the population that would not only benefit from increased research, but also requests more information about the outcomes of cannabis use (Bobitt et al., 2019).

This review will outline how cannabis influences the myelination process in the CNS by examining its impact on different cell types, and will discuss how cannabis may alter the relationships between neurons and glial cells, particularly in the aged brain. The aim of this review is to highlight the potential for cannabis to modulate myelination in the aged brain and to emphasize the paucity of research in this area in order to stimulate future research.

### 1.1. The importance of myelin

**Figure 1:**
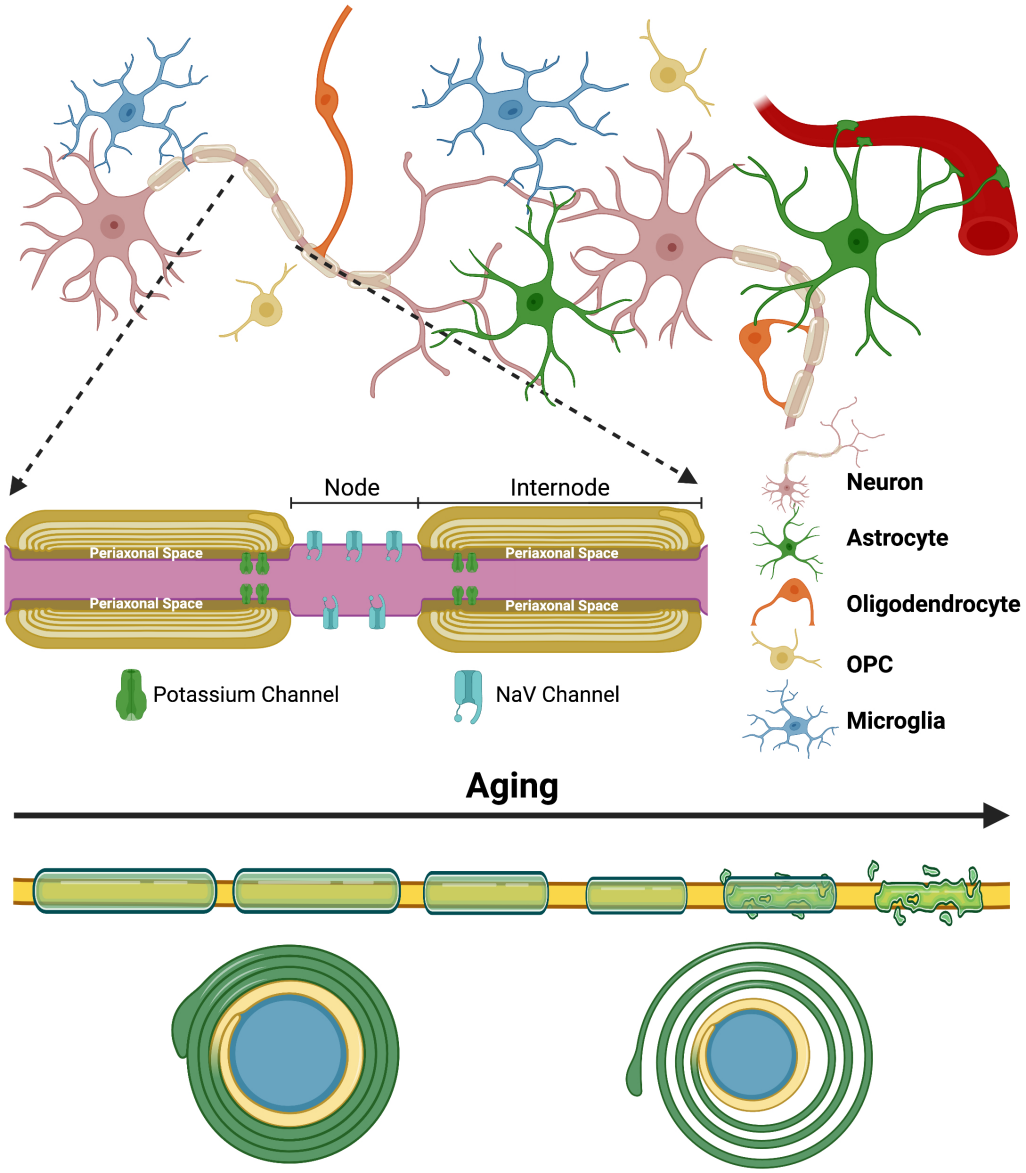
The complex glial interactions that promote proper myelination and the general impact aging has on the myelin sheath | The process of myelination is extremely complex and is constantly evolving to our experiences and various environmental insults throughout the lifespan. Myelination is highly adaptive and is fine-tuned to these experiences through neuronal activity. Proper functioning of the myelinating glial cells of the CNS—oligodendrocytes—and their precursor cells—oligodendrocyte progenitor cells (OPCs)—is essential, but the process also heavily relies on astrocytes and microglia. The top half of this figure depicts the complex arrangement that exists between the various glial cells and neurons that all contribute to proper myelination and circuit formation. The inset depicts the internodes of a myelinated axon and the nodes of Ranvier that they create. Lastly, the bottom of this figure shows the general alterations and ultimate degeneration that many myelinated axons face with increasing age. Typically, myelin sheaths become thinner, shorter and less compact (shown by the axon cross-section at the bottom) with age, although many other abnormalities also occur. These abnormalities also contribute to the disorganization of ion channels at the paranode and at the nodes of Ranvier. Created with BioRender.com.

In 1854, Rudolf Ludwig Virchow coined a term for a ubiquitous substance in the brain—myelin (Boullerne, 2016). It would take another 100 years before the central function performed by this lipid-rich sheath—saltatory conduction—was agreed upon. The rapid propagation of electrical information within the myelinated axon during saltatory conduction is possible due to the insulating properties of myelin and the nodes of Ranvier (Huxley and Stämpeli, 1949). The nodes of Ranvier are unmyelinated sections of the axon that have a high density of voltage-gated sodium (Na_v_) channels that respond rapidly to alterations in charge and function to propagate signals necessary for the depolarization of the next node during an action potential (Arancibia-Carcamo and Attwell, 2014). Myelin is therefore deposited around the axon in sections, known as internodes, which creates the boundaries for the nodes of Ranvier (Figure 1). The internodes effectively insulate the axon by increasing the resistance of the axonal membrane and by reducing the capacitance of the axon (Bakiri et al., 2011; Stadelmann et al., 2019).

Mounting evidence indicates that myelination does not become fixed after development, but is experience-driven and remains adaptive well into adulthood (Young et al., 2013; Bechler et al., 2015; Fields, 2015; Ford et al., 2015; Hill et al., 2018; Hughes et al., 2018; Jünemann et al., 2022). Various structural modifications can alter the efficiency of this system. For example, the density of Na_v_ channels within the node and the length of the node itself can both alter conduction velocity with minimal energy expenditure (Arancibia-Cárcamo et al., 2017). The diameter of the axon, thickness of the myelin sheath, and the length of the internode can also adjust conduction velocity; with wider axons, thicker sheaths, and longer internodes increasing conduction velocity up to a certain point (Waxman, 1980; Wu et al., 2012; Chapman and Hill, 2020). A large determinant of this adaptability is experience-driven neuronal activity, which has been shown to promote myelination and contribute to the modification of established myelin sheaths (Wake et al., 2011; Gibson et al., 2014; Bechler et al., 2018; Faria et al., 2019). These modifications then alter conduction speed, translating into variations in synapse strength and, therefore, synaptic plasticity (Fields, 2015).

The structural features of the myelin sheath are also important for maintaining the synchronization of action potentials within and between neurons. This synchronization is fundamental to circuit function and proper cognition. Experience-induced changes in myelination can alter this synchronicity and synaptic plasticity to optimize processing time within the circuit contextually, contributing to complex cognitive processes like social behavior, memory, motor learning and sensory experience throughout the lifespan (Liu et al., 2012; Mount and Monje, 2017; Pan et al., 2020). However, the long-range projection neurons that make up the bulk of these white matter pathways are the most vulnerable neurons during aging, and their deterioration can result in negative effects on the aforementioned cognitive processes (Mattson and Magnus, 2006).

#### 1.1.1. Myelin in the aged brain

It is now apparent that myelination does not peak until mid-life, with peak white matter volume occurring between 30 and 50-years of age in humans (Bartzokis et al., 2001; Sowell et al., 2003; Westlye et al., 2010; Buyanova and Arsalidou, 2021). Non-invasive neuroimaging techniques like diffusion tensor imaging (DTI) have been widely utilized to determine the state of white matter in the human brain throughout the lifespan. These studies have found a significant reduction in white matter volume and alterations in the integrity of myelin that suggest deterioration during aging (Pakkenberg and Gundersen, 1997; Guttmann et al., 1998; Liu et al., 2017; Vinke et al., 2018; Faizy et al., 2020). Electron microscopy performed in aged non-human primates and rodents have further clarified this deterioration by revealing the structural alterations present in the myelin sheath, such as redundant myelination and reduced myelin thickness (Figure 1; Peters, 2009; Shepherd et al., 2012; Attia et al., 2019; Phillips et al., 2019). These alterations also contribute to the disorganization of ion channels at the nodes of Ranvier and on the axonal membrane at the paranode—sections of the internode directly adjacent to the node—which likely have negative consequences on signal transduction (Hinman et al., 2006).

The macro-scale loss and micro-scale alterations in myelin impact the conduction speed and synchronization of action potentials, ultimately causing latencies and overall disruptions of neuronal communication that likely contribute to driving cognitive deficits in the aging population (Bartzokis, 2004; Bowley et al., 2010; Attia et al., 2019). Cortical disconnection due to myelin loss has been proposed as a likely candidate for reduced cognition in aged individuals for decades. For example, early *in vivo* evidence in support of this hypothesis using DTI concluded that aged individuals (56–85-years of age) show an age-related decline in white matter, especially in the frontal lobe, which was linked with impairments in executive functioning (O’Sullivan et al., 2001). This observed decline has been confirmed by more recent studies showing impairments in memory, processing speed, attention, and general cognition, which were linked to reduced myelin content (Brickman et al., 2012; Cremers et al., 2016; Coelho et al., 2021). It is important to keep in mind that these alterations occur in the “normal” aging brain, but are exacerbated in neurodegenerative diseases like Alzheimer’s disease (Papuć and Rejdak, 2020). Normal, or healthy, aging is an ill-defined term referring to the natural aging process that is devoid of significant physical or cognitive impairments and allows for the maintenance of subjective well-being (Wong, 2018). However, non-debilitating impairments are present in healthy aging, and may contribute to the progression of more serious disabilities.

**Figure 2:**
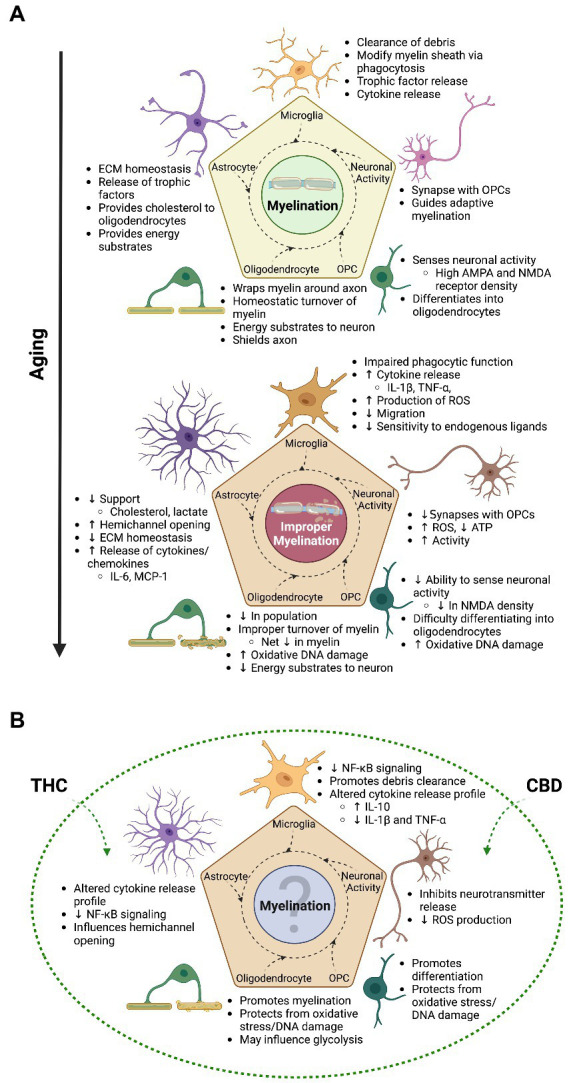
The transitional relationship between myelination and aging and the possible therapeutic advantage of cannabinoids | As emphasized throughout this review, the process of myelination is an intricate undertaking that involves all glial cells in the brain and is mainly driven by experience induced neuronal activity in adulthood and during aging. **(A)** The top panel of this figure shows some of the general contributions glial cells and neurons make to the overall process of myelination. With increasing age, a switch in cell state takes place in glial cells and neurons, ultimately leading to improper myelination and impairments in cognition. **(B)** The bottom panel showcases some of the benefits that cannabis (mainly focusing on THC and CBD) can have on myelination, as found in animal studies; with the majority looking at younger time points. Although evidence suggests improved myelination and cognition from cannabis use in aging animals, the paucity of studies focusing on this time point translates to relatively unknown overall effects of cannabis on myelination. This question mark indicates that topic, which is in need of increased research. Not only will this help fill in the gap of knowledge as to how cannabis impacts myelination across the lifespan, but will also better inform researchers on the effects of the endocannabinoid system on the aged brain. With this understanding, we are also better able to inform the public and health authorities on the impact cannabis use has on the senior population, who at the moment show increasing levels of consumption. Created with BioRender.com.

The mechanisms underlying the natural degradation of myelin and the insufficiency of remyelination in the aged brain have not yet been definitively identified. However, it is probable that there are numerous confounding factors, of which many are expected to be tightly associated with the glial cells responsible for the deposition, maintenance and modification of the myelin sheath, as discussed below (Figure 2A).

### 1.2. Oligodendrocytes and oligodendrocyte progenitor cells

In the CNS, oligodendrocyte processes wrap around axons and form the myelin sheath (Boullerne, 2016). These glial cells are capable of extending many different processes to wrap multiple axons at a time, with the ability to differentially alter the parameters of each extension due to the activity of each individual neuron contacted (Chong et al., 2012; Yeung et al., 2014). Furthermore, mature oligodendrocytes are capable of facilitating remyelination after damage in cats and non-human primates, while maintaining established myelin sheaths (Duncan et al., 2018). One particularly relevant finding is that oligodendrocytes preferentially myelinate active axons in development and adulthood, as found in primary cell cultures, zebrafish and mice (Gibson et al., 2014; Hines et al., 2015; Wake et al., 2015; Mitew et al., 2018; Faria et al., 2019). Furthermore, through DTI, neuronal circuits activated during a task were found to have increased levels of myelination in seniors, showing the ability for activity-dependent myelination in the aged human brain (Scholz et al., 2009; Jünemann et al., 2022).

The myelin sheath further provides a channel for metabolic support to the axon. Oligodendrocytes transport various energy metabolites like lactate from their soma through distinct cytoplasmic channels to the innermost layer of the myelin sheath where they are deposited into the periaxonal space (Figure 1; Fünfschilling et al., 2012; Meyer et al., 2018). The subsequent uptake of metabolites supports axon function and is essential for neuronal survival (Lee et al., 2012; Meyer et al., 2018).

However, before an oligodendrocyte can mature and participate in myelination, a complex differentiation process from oligodendrocyte progenitor cell (OPC) must occur. These cells not only populate the developing brain, but remain present in the adult brain, providing a reserve for the replacement of the mature oligodendrocyte population (Bergles and Richardson, 2016). On top of this, OPCs were shown to form direct contacts with neuronal synapses, to participate in synaptic engulfment, and to engage in axon pruning during development and adulthood, thus contributing to circuit formation and modulation in mice (Bergles et al., 2000; Auguste et al., 2022; Buchanan et al., 2022). Neuronal activity conveyed through these synapses relays information to OPCs that contributes to determining their proliferation, migration and differentiation, as well as oligodendrocyte survival, and myelination as a whole (Mitew et al., 2018; Moura et al., 2022).

#### 1.2.1. Oligodendrocytes and OPCs in the aged brain

The degeneration and improper renewal of myelin in the aged brain is predictably associated with the oligodendrocyte population. Many of the extrinsic factors that impact oligodendrocyte function [e.g., pro-inflammatory cytokines, reactive oxygen species (ROS)] are the products of other glial cells and will be discussed in more detail subsequently. Changes in axonal signaling can also present challenges for continued myelination. As described, neuronal activity induces myelination—therefore, it is likely that altered activity due to neuronal dysfunction may lead to changes in activity-dependent myelination (Sams, 2021). Furthermore, mitochondrial dysfunction in the neurons of aged mice (12-month-old) resulted in decreased production of ATP and increased production of ROS, which may subsequently damage oligodendrocytes and OPCs over time, thereby preventing OPC differentiation and myelination (Stahon et al., 2016; Spaas et al., 2021). Although the axons remain functional and oligodendrocytes remain active in 12-month-old mice, neuronal viability will likely decline due to reduced ATP and increased production of free radicals, perhaps resulting in degeneration with increasing age (Stahon et al., 2016). Conversely, age-related degeneration of myelin, oligodendrocytes and the neuron-oligodendrocyte relationship also likely leads to a decrease in metabolic support provided by oligodendrocytes, further impacting neuronal function (Hill et al., 2018; Zhang X. et al., 2021).

In aging, oligodendrocytes and OPCs also accumulate signs of oxidative DNA (mitochondrial and nuclear) damage, a feature which is commonly found in neurodegenerative diseases like Alzheimer’s disease and MS (Tse and Herrup, 2017). Oligodendrocytes and OPCs are particularly vulnerable to oxidative stress due to their extremely high metabolic demand needed for the endogenous production of myelin and their decreased ability to deal with free radicals (French et al., 2009; Giacci et al., 2018). For example, oligodendrocyte lineage cells were shown to have only half the glutathione—a major intracellular antioxidant—content compared to astrocytes in primary cell cultures from rats (Juurlink et al., 1998). Importantly, synthesis of glutathione naturally declines during aging in mice, further rendering oligodendrocyte lineage cells susceptible to damage and dysfunction (Wang, 2003). Additionally, OPCs are particularly susceptible to damage by oxidizing agents because of a delay in the production or reduced activity of antioxidant enzymes (e.g., glutathione peroxidase), which increases with maturation (Back et al., 1998; Baud, 2004; Spaas et al., 2021). A recent review hypothesizes that this increased vulnerability to oxidative stress may inhibit OPC differentiation (Spaas et al., 2021).

A decline in newly formed mature oligodendrocytes has been observed in the aged brain (Soreq et al., 2017; Hill et al., 2018; Wang et al., 2020; Rivera et al., 2021; Dimovasili et al., 2022). However, OPC density does not seem to decline with age when compared across the lifespan in mice, non-human primates or humans (Sim et al., 2002; Doucette et al., 2010; Yeung et al., 2014; Wang et al., 2020; Dimovasili et al., 2022). Although it is generally accepted that the OPC population remains stable in the aged brain, a more recent study found significantly reduced OPC (NG2^+^) density in the corpus callosum of 18-month-old mice (Rivera et al., 2021). Nonetheless, the inability of OPCs to differentiate into mature oligodendrocytes in the aged brain is not currently debated, and it likely contributes to the reduced capacity for remyelination and declining oligodendrocyte population (Sim et al., 2002; Neumann et al., 2019; Segel et al., 2019; Rivera et al., 2021; Zhang X. et al., 2021; Dimovasili et al., 2022). This reduced ability to differentiate may be a consequence of a markedly different proteome in aged OPCs (de la Fuente et al., 2020). For example, aldehyde dehydrogenase 1 family member A1 (ALDH1A1) and transcription factor 4 (TCF4) are both involved in OPC differentiation and have notably reduced expression levels in aged rats (>15-months-old; de la Fuente et al., 2020).

OPCs also display differing levels of ion channels and receptors depending on age and brain region (Spitzer et al., 2019). For example, the density of N-methyl-D-aspartate (NMDA) receptors on OPCs significantly declines with increasing age (>6-months-old) in multiple regions of the mouse brain, including the corpus callosum (Spitzer et al., 2019). This change likely affects activity-dependent myelination in the aged brain by significantly impacting the ability for OPCs to sense and act on glutamate released from active neurons (Gautier et al., 2015; Spitzer et al., 2019). Interestingly, this NMDA receptor-mediated activity-dependent myelination requires the simultaneous presence of glutamate and growth factors like brain derived neurotrophic factor (BDNF), hinting at the importance of surrounding glial cells (Lundgaard et al., 2013). As a note, α-amino-3-hydroxy-5-methyl-4-isoxazolepropionic acid (AMPA)/kainate receptors are important for the initial stages of remyelination after experimentally induced demyelination, where the binding of glutamate released from active axons within the lesion promotes proliferation, survival and differentiation of OPCs during development and adulthood in rodents (Gautier et al., 2015; Kougioumtzidou et al., 2017). Although AMPA/kainate receptor levels remain relatively constant or increase with age, the impaired ability for neurons to form new synaptic contacts with OPCs, combined with a decline in NMDA receptors, may decrease OPC differentiation and activity-dependent myelination in the aged brain (Spitzer et al., 2019; Sams, 2021).

A general increase in ROS and free radicals in the aged brain and a phenomenon known as “niche stiffening” of the extracellular matrix (ECM) directly adjacent to OPCs can also prevent their differentiation (French et al., 2009; Segel et al., 2019). Niche stiffening is a dynamic process that physically alters the elasticity of tissue, which increases the rigidness of the ECM in the brain of aged mice (>14-months-old; Swift et al., 2013; Segel et al., 2019). The mechanosensitive ion channel PIEZO1 was found to be essential for the OPC detection of the elasticity of the ECM, and the knockdown of PIEZO1 resulted in increased proliferation and differentiation of OPCs in the aged CNS of mice (Segel et al., 2019). Furthermore, the introduction of aged OPCs into the ECM within the prefrontal cortex of neonatal rats rescued their proliferative and differentiating abilities, indicating the importance of the environment for these OPC functions (Segel et al., 2019).

Microglia and astrocytes may also influence OPC cell fate through their modulation of the ECM. For instance, primary microglial cells from aged (18–24-months-old) rats treated with an inflammatory stimulus [i.e., transforming growth factor-β (TGF-β)] creates a microglial-deposited ECM that promotes the differentiation of OPCs into astrocytes, thereby preventing oligodendrocyte differentiation and myelination (Baror et al., 2019). Furthermore, oligodendrocytes in aged mice (18-months-old) release factors that promote microglial-mediated survival of oligodendrocytes, but prevent OPC differentiation (Luan et al., 2021). Similarly, astrocytes were found to inhibit OPC differentiation and disrupt remyelination by releasing chondroitin sulfate proteoglycans into the ECM in primary mixed glial cell cultures from mice (Keough et al., 2016).

Lastly, as mentioned, neurotrophic factors (e.g., BDNF) released from glial cells impact the ability for OPCs to contribute to activity-dependent myelination. These are just a few examples of the influence surrounding glial cells have on OPCs, oligodendrocytes and the process of myelination. However, astrocytes and microglia are profoundly altered structurally and functionally in the aged brain, and these changes extend to the cells and structures they support (Figure 2A).

### 1.3. Astrocytes in myelination

Astrocytes are an extremely diverse group of glial cells that contribute to the blood–brain barrier (BBB), blood flow modulation, metabolite supply, and perform modulatory roles at the synapse involved in synaptic activity and plasticity (Sofroniew and Vinters, 2010). Furthermore, astrocytes are vital for myelination. The loss of astrocytes reduces the density of oligodendrocytes, initiates demyelination, and promotes myelin abnormalities (e.g., decompaction) in the white matter of developing and adolescent mice (7 days to ~1.5-months-old; Tognatta et al., 2020). These effects are likely partly due to local increases in extracellular glutamate causing excitotoxicity, and reduced trophic support [e.g., platelet-derived growth factor (PDGF)] from astrocytes (Tognatta et al., 2020). The uptake of glutamate from the extracellular space is an essential function performed by astrocytes that prevents excitotoxicity of neurons and glial cells (Mcdonald et al., 1998; Hassel et al., 2003; Matute et al., 2007; Goursaud et al., 2009; Mahmoud et al., 2019). Additionally, the controlled release of gliotransmitters (e.g., glutamate) from astrocytic hemichannels—membrane channels between cells and the extracellular space made up of connexin proteins—directly to synapses modulates synaptic transmission and plasticity, and was even shown to be essential for behavioral outputs including fear memory consolidation in rats (~2-months-old; Ye et al., 2003; Stehberg et al., 2012; Abudara et al., 2018).

Astrocytes also maintain appropriate levels of K^+^ ions in the extracellular space and effectively disperse them throughout the pan-glial network that spans the entire brain (Beckner, 2020). The pan-glial network allows for the diffusion of ions and small metabolites between coupled cells connected through gap junctions, which are made up of adjoining hemichannels (Orthmann-Murphy et al., 2008; Stephan et al., 2021). The loss of astrocyte-oligodendrocyte gap junctions results in myelin pathology (e.g., vacuolation) and loss of astrocytes (Magnotti et al., 2011; Tress et al., 2012). Additionally, many human diseases characterized by demyelination (e.g., MS and neuromyelitis optica) show early disruption of gap junctions between astrocytes and oligodendrocytes and a decline in connexin proteins (Markoullis et al., 2012; Masaki, 2015).

Astrocytes are also a main source of cholesterol, facilitate iron transport, and directly provide oligodendrocytes with metabolic support (*via* gap junctions) in the adult CNS, all contributing to the processes needed to synthesize myelin (Jurevics and Morell, 2002; Schulz et al., 2012; Saher and Stumpf, 2015; Camargo et al., 2017; Cheli et al., 2020). Lastly, a wide range of soluble factors [e.g., BDNF and chemokine (C-X-C motif) ligand 1 (CXCL1)] released by astrocytes can have myriad effects on myelination, as found in rodents (Tsai et al., 2002; Fulmer et al., 2014; Kıray et al., 2016).

#### 1.3.1. Astrocytes and myelination in the aged brain

Due to the vast number of functions performed by astrocytes in the CNS, age-related dysfunction of these cells predictably has wide-ranging impacts on brain function. Firstly, observations in aged mice (20–24-months-old) reveal morphological changes in astrocytes and alterations in territorial domain that may result in reduced contacts between adjacent astrocytes, disconnecting them from the greater pan-glial network which is essential for many homeostatic functions (e.g., K^+^ spatial buffering, metabolic and cholesterol supply to oligodendrocytes; Grosche et al., 2013; Popov et al., 2021; Verkhratsky et al., 2022). A steady reduction in astrocytic glutamate transporters, reduced capacity to buffer and disperse K^+^, and an overall decrease in their ability to sense synaptic activity (partly due to reduced density of ionotropic receptors) was also observed in aged mice (20–24-months-old), disrupting long-term potentiation of synapses in the hippocampus (Lalo et al., 2011; Popov et al., 2021). This is likely partly due to excess levels of glutamate in the extracellular space resulting in excitotoxicity, which damages neurons, oligodendrocytes, and myelin (Olney, 1971; Matute et al., 2007; Fu et al., 2009). Additionally, the increase in hemichannel activation due to an increase in pro-inflammatory cytokines [e.g., tumor necrosis factor-α (TNF-α) and interleukin-1β (IL-1β)] in primary cell cultures and mice results in an increase in the release of various ions (i.e., K^+^, Ca^2+^) and gliotransmitters (i.e., ATP, glutamate) into the extracellular space, further disrupting homeostasis and possibly contributing to neuronal death (Retamal et al., 2007; Froger et al., 2010; Karpuk et al., 2011; Orellana et al., 2011; Satarker et al., 2022). The cholesterol synthesis pathway is also significantly altered in astrocytes from aged mice (24-months-old), likely contributing to the observed decline of cholesterol in the aged brain and potentially hindering production of myelin (Boisvert et al., 2018; Palmer and Ousman, 2018).

An altered gene expression profile is further observed in aged astrocytes. Increases in genes associated with cytokine pathways, antigen presentation, the complement cascade, and reactivity [e.g., glial fibrillary acidic protein (*Gfap*)] were observed in astrocytes in the hippocampus, hypothalamus, visual cortex, striatum and cerebellum of aged mice (24-months-old; Boisvert et al., 2018; Clarke et al., 2018). GFAP is an intermediate filament protein commonly used as a marker for astrocytes, which significantly increases in pathological-like states (e.g., aging and MS), indicating heightened astrocyte reactivity in rodents and humans (Nichols et al., 1993; Saraste et al., 2021).

This elevated expression of GFAP has also been linked to astrocyte senescence (Salminen et al., 2011; Boisvert et al., 2018). Senescence refers to cells that enter into a distinct state characterized by dysfunctional mitochondria, increased production of ROS, and an altered secretory profile as a consequence of DNA damage, telomere shortening and an altered environment (Sikora et al., 2021). The downstream effects of these pathways exacerbate inflammation and impair myelination *via* inhibition of the OPC cycle and loss of functional support for oligodendrocytes by astrocytes, as shown in primary cell cultures and in mice (Palmer and Ousman, 2018; Willis et al., 2020). Interestingly, astrocytes often do not transition into this more damaging/senescent-like state unless microglia induce the switch through the release of cytokines like IL-1α and TNF (Herx et al., 2000; Liddelow et al., 2017; Clarke et al., 2018; Jha et al., 2019). There are numerous reasons for an altered microglial secretory profile in aging, but one specific to white matter regions could be an increase in myelin debris that cannot be efficiently cleared/metabolized by microglia, resulting in cellular stress (Safaiyan et al., 2016).

### 1.4. Microglia in myelination

Microglia—the resident immune cells of the CNS—perform vital functions in all stages of life. They act as gardeners, constantly surveying their surroundings looking for debris to clear, shaping and pruning synapses, maintaining appropriate glial and neuronal population sizes, modulating neuronal activity, and releasing various trophic factors to support growth and development of glial cells and neurons (Šimončičová et al., 2022). Microglia also play a substantial role in myelination, contributing to the developmental and experience-driven process of adaptive myelination (Kalafatakis and Karagogeos, 2021; Santos and Fields, 2021). In fact, microglia are present at higher densities in human white matter compared to gray matter, highlighting their importance in this environment (Mittelbronn et al., 2001; Askew et al., 2017). However, evidence is more contradictory for microglial density in mice. One study found increased density of microglia in the white matter of the forebrain, whereas an earlier study found increased density in gray matter from the entire mouse brain (Lawson et al., 1990; Savchenko et al., 2000). Therefore, it is important to keep in mind the region analyzed, as microglial density and function can greatly vary. Additionally, it is important to note that the microglial population is not homogenous, but instead exists as a continuum of states that contribute in divergent fashions to supporting brain development, activity, plasticity and integrity (Paolicelli et al., 2022).

Although microglia have a plethora of functions, three of their activities primarily contribute to myelination. (1) Microglia release a repertoire of soluble factors [e.g., insulin growth factor-1 (IGF-1), IL-1β, TGF-β] that facilitate the promotion and prevention of myelination (Hsieh et al., 2004; Pang et al., 2007; Santos and Fields, 2021; McNamara et al., 2023). (2) Microglia phagocytose myelin debris, which is important as myelin debris can inhibit OPC differentiation, while efficient clearance of myelin debris allows for effective remyelination after experimental demyelination in rodents (Kotter, 2006; Neumann et al., 2008; Lampron et al., 2015). It was also reported that microglia are capable of removing incorrectly deposited myelin directly from the axon, contributing to the refinement of myelin sheaths, as shown in zebrafish and mice during development (Hughes and Appel, 2020; Djannatian et al., 2023). (3) Microglia dynamically contact active axons, guided by the nodal efflux of K^+^ ions. This interaction was associated with improved remyelination after experimental demyelination in mice, and may be a way by which microglia prevent neuronal damage from hyperactivity (Madry et al., 2018; Ronzano et al., 2021). Microglia respond rapidly to hyperactive neurons and wrap their processes around axons to facilitate rapid repolarization, thus preventing excitotoxicity and maintaining neuronal viability in mice (Kato et al., 2016). Therefore, microglia are emerging as essential modulators of neuronal activity that substantially contribute to determining neuronal architecture and function (Badimon et al., 2020; Cserép et al., 2021).

#### 1.4.1. Microglia and myelination in the aged brain

Microglia are not immune to the challenges of aging. Changes observed in aged mice (≥12-months-old) include an upregulation of genes associated with the immune response, and a decrease in genes associated with environment probing and interactions with the ECM (Grabert et al., 2016; Angelova and Brown, 2019). Furthermore, an age-related metamorphosis in their secretory profile, resulting in increased pro-inflammatory markers is also a common characteristic of microglia, as observed in those sorted from the aged mouse brain (≥18-months-old; Holling et al., 2004; Sierra et al., 2007; Norden and Godbout, 2013; Koellhoffer et al., 2017; Marschallinger et al., 2020). For instance, aged mice have microglia with increased levels of the nod-like receptor protein 3 (NLRP3) inflammasome (Youm et al., 2013). After inflammasome activation, an increase in the production of IL-1β, IL-6, TNF-α and others are observed in the mouse brain (Youm et al., 2013; Hu et al., 2019). Many of these compounds are beneficial and required for proper myelination, however, a problem arises when this activation becomes chronic, as is the case in the aged brain (Tilstra et al., 2011). Prolonged activation of inflammatory pathways in astrocytes and microglia promote demyelination, while their inhibition has the potential to support remyelination in rodents (Jha et al., 2010; Raasch et al., 2011; Goldmann et al., 2013; Blank and Prinz, 2014).

Impairments in microglial phagocytosis of cellular debris is common in the aged mouse brain (≥20-months-old) as well, perhaps contributing to the increase in inflammatory factors seen during aging (Ritzel et al., 2015; Safaiyan et al., 2016; Cantuti-Castelvetri et al., 2018; Marschallinger et al., 2020). Impaired phagocytosis could be partly attributed to the dysfunction of an overwhelmed clearance system (Safaiyan et al., 2016; Thériault and Rivest, 2016; Marschallinger et al., 2020). For instance, continuous increases in myelin debris cannot be accommodated by microglia in the long-term, which subsequently leads to dysfunctional lysosomal activity and a noticeable increase in insoluble lipofuscin-like granules—a marker of aging, dystrophy and possibly senescence—in mice (Safaiyan et al., 2016).

A specific population of microglia in the white matter of the aged brain (≥18-months-old), white matter associated microglia (WAM), has an increased expression of triggering receptor expressed on myeloid cells 2 (TREM2)—a receptor important for phagocytosis, lipid metabolism and proper myelination (Poliani et al., 2015; Safaiyan et al., 2021). In aged humans (50–80-years-old), microglia in the white matter have increased expression of genes associated with lipid-metabolism as well [e.g., secreted phosphoprotein 1 (SPP1) and apolipoprotein E (APOE)], hinting at the possibility of the presence of WAMs in humans; although TREM2 did not significantly associate with these white matter microglial clusters (Sankowski et al., 2019; Safaiyan et al., 2021). Their function is likely similar to the hypothesized function of WAMs in mice, which is thought to include myelin debris clearance and lipid metabolism (Safaiyan et al., 2021).

Interestingly, microglia isolated from whole brains of aged mice (≥21-months-old) show significantly downregulated expression levels of TREM2 compared to younger mice (Hickman et al., 2013; Thomas et al., 2022). This could be due to a net decrease in microglial expression of TREM2, despite the increases seen in WAM. However, it has also been hypothesized that with time WAMs may become overwhelmed and enter into a senescent state, thereby reducing function and contributing to white matter degeneration and cognitive decline (Ahn et al., 2022). This senescent state—perhaps more common in WAM from rodents >18-months of age—may have reduced expression of TREM2, contributing to the observed decline in expression with age.

Senescent microglia may have a reduced capacity to modulate neuronal activity because of genetic changes that result in reduced surveillance, migration, and sensitivity to endogenous ligands, and a heightened sensitivity to pathogens, as seen in 24-month-old mice (Hickman et al., 2013; Madry et al., 2018; Angelova and Brown, 2019). For example, due to the downregulation of genes for purinergic receptors (e.g., *P2ry12*) and potassium leak channels (e.g., *Thik-1*) in aged mice, the ability for microglia to sense hyperactive neurons and migrate toward them in order to facilitate rapid repolarization would be reduced, impairing their beneficial modulation (Hickman et al., 2013; Kato et al., 2016; Madry et al., 2018). This may be a contributing factor for neuronal hyperactivity and excitotoxicity found in the aged brain, which negatively affects memory (Bishop et al., 2010; Stargardt et al., 2015; Li et al., 2020).

The age-mediated alterations in microglial function that are evident in the aged brain are thought to arise from at least two factors. (1) Microglia become overwhelmed and cannot keep up with demand, and/or (2) shift into a less sensitive state trying to keep inflammatory signals to a minimum by reducing their reactivity to endogenous ligands (Hickman et al., 2013). Although some microglia may enter into a senescent state, many of these cells still have the ability to aid the aged brain and maintain/improve cognition. An interesting avenue to achieve this may be through the use of cannabis and the endocannabinoid system. Indeed, all glial cell types have receptors for cannabinoids, which have wide ranging effects on cellular function, indicating that cannabis may be beneficial for glial regulation of myelination (Figure 2B; Stella, 2010; Navarrete et al., 2014; Ilyasov et al., 2018; Martinez Ramirez et al., 2023).

### 1.5. The endocannabinoid system

The endocannabinoid system extends to most regions of the body. It encompasses naturally occurring endogenous (endo)cannabinoids, the enzymes needed for their formation and degradation, and cannabinoid receptors (Lu and Mackie, 2016). Although many receptors take part in endocannabinoid signaling [e.g., peroxisome proliferator-activated receptors (PPARs), transient receptor potential cation channels (TRPs)], the two main cannabinoid receptors are cannabinoid receptor type 1 (CB_1_R) and cannabinoid receptor type 2 (CB_2_R; Howlett, 2002).

CB_1_Rs are widespread in the CNS and are the most prevalent G-protein coupled receptor in the mammalian brain (Herkenham et al., 1990; Marsicano and Lutz, 1999). In the hippocampus and cortex, CB_1_Rs have an especially high localization on inhibitory neurons, although their distribution and localization patterns differ throughout the human and rodent CNS (Glass et al., 1997; Marsicano and Lutz, 1999; Tsou et al., 1999; Fletcher-Jones et al., 2020). Interestingly, a significant number of CB_1_Rs are not expressed on the cell surface, but instead localize to lysosomes and late endosomes, as shown in cell lines and primary cell cultures—possibly contributing to lysosomal integrity and function (Rozenfeld and Devi, 2008; Bilkei-Gorzo, 2012; Fletcher-Jones et al., 2020). Additionally, CB_1_Rs also localize to mitochondria and can influence metabolism in neurons and glial cells, as observed in primary cell cultures and in mice (Bénard et al., 2012; Jimenez-Blasco et al., 2020).

However, the majority of neuronal CB_1_Rs are found on pre-synaptic terminals, where their primary function is to suppress the release of neurotransmitters, altering the activation of post-synaptic channels and, therefore, modulating synaptic activity and plasticity (Mackie and Hille, 1992; Di Marzo et al., 2015; Zou and Kumar, 2018). The cannabinoid-mediated reduction in neurotransmitter release is achieved by the reduced influx of presynaptic Ca^2+^ due to the inhibition of voltage gated Ca^2+^ channels and of adenylyl cyclase, which downregulates cyclic adenosine monophosphate and protein kinase A, two cellular constituents involved in increasing the influx of Ca^2+^ (Castillo et al., 2012). CB_1_R activation in pre-synaptic terminals is mainly facilitated through retrograde signaling of endocannabinoids released from the post-synapse (Kano et al., 2009; Castillo et al., 2012; Njoo et al., 2015).

CB_2_Rs have much lower levels of expression in the CNS of humans and rodents (Lu and Mackie, 2016; Jordan and Xi, 2019). Interestingly, CB_2_R mRNA expression can vastly increase during an inflammatory insult, with microglia from mice displaying as much as a 10-fold increase (Maresz et al., 2005). This finding indicated that the CB_2_R likely plays a substantial role in CNS immune function, which has been subsequently supported in the literature (Turcotte et al., 2016; Komorowska-Müller and Schmöle, 2020). CBRs may attenuate pro-inflammatory cytokine secretion by interfering with the phosphorylation of mitogen activated protein kinases (MAPK), such as extracellular signal-regulated kinase (ERK), which is known to participate in pro-inflammatory pathways, as shown in microglial cell line cultures (Eljaschewitsch et al., 2006; Young and Denovan-Wright, 2022a,b). Furthermore, activation of CB_2_Rs promotes IL-10 (an anti-inflammatory cytokine) secretion from primary microglia cells from mice by reducing the translocation of the transcription factor nuclear factor-κB (NF-κB) to the nucleus *via* reduced phosphorylation of IκB Kinase-α (IKKα)—a subunit of the IKK complex that is essential for NF-κB signaling—which subsequently prevents NF-κB formation (Solt and May, 2008; Correa et al., 2010). Of note, NF-κB-mediated inflammation is often *via* the inflammasome and is associated with many white matter associated diseases and is upregulated in the aged brain (Tilstra et al., 2011; Youm et al., 2013; Blank and Prinz, 2014; Rea et al., 2018). Evidence also suggests that CB_2_Rs are present on post-synaptic terminals of neurons in rodents and non-human primates, although expression levels are relatively low and may depend on brain region (Brusco et al., 2008; Lanciego et al., 2011; Li and Kim, 2015; Stempel et al., 2016).

Importantly, astrocytes, oligodendrocytes, and OPCs possess CB_1_Rs and CB_2_Rs, highlighting the wide range of functions performed by this system (Navarrete et al., 2014; Ilyasov et al., 2018; Martinez Ramirez et al., 2023). The impact these receptors have with respect to their activation by cannabinoids on individual cell-types will be discussed in more detail below (Figure 2B).

#### 1.5.1. The endocannabinoid system in aging

The endocannabinoid system undergoes an unequivocal transition during aging across species (Bilkei-Gorzo, 2012; Bishay et al., 2013; Pascual et al., 2013; Di Marzo et al., 2015; Piyanova et al., 2015). The direction of this change relies on the region investigated and, therefore, its impact on brain function differs based on the affected region and the context in which it is examined.

A general trend in the literature suggests that CB_1_R density decreases throughout the brain with aging, however, receptor function seems to differentially change depending on the region and cell type (Mato and Pazos, 2004; Bilkei-Gorzo, 2012; Di Marzo et al., 2015; Ginsburg and Hensler, 2022). Interestingly, G_i/o_-coupled protein receptors decline in the aged brain as a whole (de Oliveira et al., 2019).

The CB_2_R is less well characterized due to methodological difficulties and, therefore, the change in CB_2_R expression with age is less well-known (Zhang et al., 2019). One study did not find any reductions in CB_2_R density in any region analyzed from aged mice (22-months-old), while an earlier study found a significantly declined receptor density in synaptosomes, but not in overall membrane fractions from aged rats (24–28-months-old; Pascual et al., 2014; Hodges et al., 2020). This discrepancy could be a result of different rodent species and/or differential CB_2_R expression based on cell type. It could be speculated that neuronal synaptic expression of CB_2_Rs decline with age, whereas glial expression remains constant or potentially increases, which would warrant further investigation.

Sex differences are a common feature of the endocannabinoid system, although this depends on the type of measurement and regions analyzed (Laurikainen et al., 2019; Van Ryzin et al., 2019; De Meij et al., 2021; Levine et al., 2021; Vecchiarelli et al., 2022). For example, in the human brain, females exhibited increased binding of the CB_1_R with age, whereas males did not show any change (Van Laere et al., 2008). Similarly, adult female CB_2_R-knockout (KO) mice displayed larger alterations in synaptic markers compared to male mice, although both sexes exhibited deficits in social memory (Komorowska-Müller et al., 2021b).

Aging impacts the endocannabinoid system on multiple levels, and myelination relies on support from numerous cell types and is moderately guided by neuronal activity, two processes which are partly controlled by the endocannabinoid system. Therefore, any modification to endocannabinoid signaling will likely have an impact on myelination, one of the most important structural and functional aspects of the CNS.

### 1.6. Cannabis and the endocannabinoid system

Cannabinoids exert their influence over the endocannabinoid system mainly through CB_1_Rs and CB_2_Rs, which contribute to the sought after medicinal and recreational qualities of cannabis. The most common psychoactive cannabinoid, Δ-9-tetrahydrocannabinol (THC), has a relatively high affinity for the two cannabinoid receptors (Pertwee, 2008). THC is generally considered to be a partial agonist for both CBRs, although its inhibitory effect on synapses can be comparable to that of a full agonist (Laaris et al., 2010). By contrast, cannabidiol (CBD)—the most common non-psychoactive cannabinoid—does not have a particularly high affinity for either CBR, but was shown to antagonize CBR agonists (Thomas et al., 2007). CBD is a negative allosteric modulator of CB_1_Rs, and is suggested to act as an inverse agonist of CB_2_Rs (Thomas et al., 2007; Laprairie et al., 2015). The anti-inflammatory effects attributed to CBD may be exerted through this inverse agonism of CB_2_Rs (Thomas et al., 2007; Pertwee, 2008; Yu et al., 2020). However, it is important to note that both THC and CBD have many CBR-independent or indirect signaling mechanisms that also contribute to their overall outcomes (Pertwee, 2008; Stella, 2010). Interestingly, one indirect mechanism is the ability for CBD to inhibit fatty acid amide hydrolase (FAAH)—the enzyme required for the degradation of *N*-arachidonoylethanolamine (anandamide; AEA), an endocannabinoid—which results in an increase in AEA (Watanabe et al., 1996; Bisogno et al., 2001; De Petrocellis et al., 2011). Of note, there is a large body of research looking at the potential benefits of inhibiting endocannabinoid metabolizing enzymes; with studies showing that increases in AEA have an immunomodulatory effect, and can potentially aid in MS, as shown in mice (Rossi et al., 2010; Vázquez et al., 2015a; Vecchiarelli et al., 2021).

The changes observed in the endocannabinoid system in the aging brain are mostly similar to those observed after chronic THC exposure (Yoo et al., 2020). The most noticeable effect observed after chronic THC exposure is the significant but reversible reduction in CB_1_Rs, with cortical regions showing more extensive decreases in expression (Hirvonen et al., 2012; D’Souza et al., 2016; Augustin and Lovinger, 2022). However, it should be noted that these studies used exclusively male participants. Studies including females are lacking, which is a significant gap since sex differences with respect to the endocannabinoid system are well-described (Laurikainen et al., 2019; Levine et al., 2021).

Importantly, THC does not interact with the brain in equal measure, as found by Leishman et al. (2018). In this study, acute administration of THC [3 mg/kg; intraperitoneal injection (i.p)] differentially impacted the lipidome and transcriptome depending on the brain region and age of the subject, 2 h after administration. THC is distributed and metabolized in a region-specific manner, with highest levels in the hippocampus. Another interesting finding of this study was that adult mice (~4-months-old) displayed the largest changes after acute THC exposure compared to exposed ~1 and ~2-month-old mice, with a general downregulation of the endocannabinoid system. The effect of THC in the aged brain was not examined in this study, but the observed changes would likely be different than other time points.

Although THC and CBD are the main cannabinoids found in cannabis, it is important to note that 100 s of different cannabinoids and other biologically active compounds exist in the plant, such as terpenes, including β-caryophyllene (Kopustinskiene et al., 2022). These compounds likely work synergistically to produce the effects of cannabis through multiple different signaling pathways, creating what is called the “entourage effect” (Ferber et al., 2020; Finlay et al., 2020). However, due to a lack of literature, the next sections will focus on THC, CBD, and some synthetic cannabinoids.

### 1.7. The impact of cannabis on microglia

One of the most essential microglial functions that promotes myelination is the clearance of myelin debris (Kotter, 2006; Lampron et al., 2015). The CB_2_R has been shown to be important for phagocytosis in microglia. For instance, in CB_2_R-KO primary microglia from mice, phagocytosis was significantly reduced in both steady-state conditions and following an inflammatory stimulus (i.e., TGF-β) compared to controls (Mecha et al., 2015). Similarly, activation of the CB_2_R increased phagocytosis in primary cell cultures and improved the removal of amyloid-β_40_ peptides in a mouse model of Alzheimer’s disease pathology (Ehrhart et al., 2005; Aso et al., 2016). Additionally, microglia from CB_2_R-KO mice (18-months-old) had an age-dependent increase in lipofuscin granules compared to the control group, signifying reduced lysosomal degradation (Komorowska-Müller et al., 2021a). These findings indicate a significant role played by CB_2_Rs in the proper removal and degradation of debris, and in the microglial response to environmental stimuli. However, other receptors likely also contribute, as CBD has been shown to promote phagocytosis through TRPs in primary microglial cell cultures from mice (Hassan et al., 2014; Yang et al., 2022). Briefly, TRPs are Ca^2+^-permeable channels known for their role in temperature sensation, but interestingly, have also been shown to play a role in the release of pro-inflammatory cytokines from microglia in mice exposed to immunogenic agents [i.e., lipopolysaccharide (LPS)] (Zhang Y. et al., 2021). The observed increase in phagocytosis due to CBD is thought to be related to the TRP-mediated increase in the influx of Ca^2+^ (Hassan et al., 2014).

Increased microglial phagocytosis of myelin debris following the application of 2-AG subcutaneously *via* an osmotic pump subsequently promoted remyelination in an adult mouse model of experimental demyelination (Mecha et al., 2019). This study also observed an altered secretory profile, with increases in IL-1β, TNF-α, and IL-10 in the brain after 2-AG application. This is an example of an augmented immune response, where the benefits of pro-inflammatory cytokines in tandem with anti-inflammatory cytokines work synergistically to promote remyelination. However, microglia in the aged brain are responding to chronically elevated levels of pro-inflammatory factors resulting in reduced functional capacity.

Activation of the CB_2_R by AEA in primary microglial cells from mice has been shown to reduce NF-κB signaling and increase expression of IL-10 (Correa et al., 2010). The anti-inflammatory effect produced by IL-10 is partly through a negative feedback loop with astrocytes, where the binding of IL-10 causes the release of TGF-β from astrocytes, which subsequently attenuates pro-inflammatory cytokine production in primary microglia cells (Norden et al., 2014). However, astrocytes have reduced expression of IL-10 receptor-1 in aged mice (≥18-months-old), and fail to effectively diminish microglia-mediated inflammation (Norden et al., 2016; O’Neil et al., 2022). Therefore, compounds that can act directly on microglia to reduce pro-inflammatory cytokine production are of particular interest.

Selective CB_2_R agonists (i.e., JWH-133; i.p.) reduce the release of pro-inflammatory cytokines from microglia in a mouse model of Alzheimer’s disease pathology (Aso et al., 2013). Similarly, activation of CB_1_Rs and CB_2_Rs by synthetic cannabinoids [i.e., arachidonyl-2^′^-chloroethylamide (ACEA) and HU-308, respectively] reduced nitric oxide, TNF-⍺, IL-1β and IL-6 release from spontaneous immortalized microglia (SIM)-A9 cells in culture (Young and Denovan-Wright, 2022a).

CBD also has the ability to beneficially regulate the oxidative status in microglia by acting as an antioxidant, where it may directly scavenge ROS and/or inhibit the phosphorylation of upstream kinases needed for NF-κB signaling in primary microglia cells cultured from mice (van den Berg et al., 2001; dos-Santos-Pereira et al., 2020; Atalay Ekiner et al., 2022). This results in reduced levels of IL-1β and TNF-α independently of CB_1_R, CB_2_R or PPARγ, as tested using receptor antagonists. These effects could also be partly regulated by increased levels of endocannabinoids or through TRP channels, since CBD has a relatively high affinity for TRP vanilloid receptor 1 (TRPV1), which has been shown to modulate cytokine production in microglia (Stampanoni Bassi et al., 2019). Furthermore, in a mouse (1-month-old) model of viral-induced demyelination, CBD (5 mg/kg; i.p) attenuated morphological alterations in microglia, and reduced production of IL-1β, chemokines, and vascular cell adhesion molecule-1 (VCAM-1) through adenosine A_2A_ receptors (Mecha et al., 2013). Interestingly, VCAM-1—a protein expressed by endothelial cells in the BBB that is involved in peripheral immune cell recruitment—expression increases in the aged brain, and the application of anti-VCAM-1 antibodies reduces microglial reactivity and improves memory and learning in aged (19-month-old) mice (Yousef et al., 2019). Overall, it is clear that CBD acts through a number of vastly different pathways that have overlapping effects on the brain.

Although the anti-inflammatory effects of the CB_2_R are well-characterized, it is also important to note that the CB_2_R seems essential for many environmental-induced immune responses in microglia. Primary microglia cell and organotypic hippocampal slice cultures generated from CB_2_R-KO mice showed attenuation of the microglial immune response to toll-like receptor (TLR) ligands (e.g., TLR4/3/9), preventing the pro-inflammatory cascade usually associated with TLR ligands (Reusch et al., 2022). Therefore, although the CB_2_R can function to suppress inflammation, it also contributes to its initiation. These studies highlight how complex the interaction between the endocannabinoid system and microglia is.

The CB_1_R is also important for the inflammatory response, as a recent study found that inflammation was dependent on microglial CB_1_Rs (De Meij et al., 2021). They found decreased pro-inflammatory cytokines in mice (2–5-months-old) with CB_1_R-KO microglia exposed to LPS. However, it also increased sickness behavior in male, but not female mice (De Meij et al., 2021). Similarly, ablation of the CB_1_R resulted in early age-related cognitive deficits in mice (Bilkei-Gorzo et al., 2005; Albayram et al., 2011). Therefore, the activation of CB_1_Rs may be beneficial for preventing sickness behavior and age-related cognitive decline. This is true with respect to THC, but only with certain doses (Sarne, 2019). Ultra-low (0.002 mg/kg; i.p) and low (3 mg/kg; i.p) doses of THC resulted in improved cognitive function in old mice (24 and 18-month-old, respectively), whereas the same dose induced cognitive impairments in adult mice (2-months-old; Bilkei-Gorzo et al., 2017; Sarne et al., 2018). However, higher doses of THC have the reverse effect (Calabrese and Rubio-Casillas, 2018). This dose-dependent alteration was also observed in microglia in the 2-month-old mouse brain, where higher doses (20 mg/kg; i.p) of THC resulted in the increased release of pro-inflammatory cytokines compared to lower doses (Cutando et al., 2013). In line with this, a recent study found that adolescent mice exposed to daily low-doses of THC (5 mg/kg; i.p) resulted in the downregulation of genes responsible for the microglial response to an immune insult (e.g., IL-1β, IL-6), which carried over into young adulthood (~1.5-months-old) but not maturity (~4-months-old; Lee et al., 2022). This blunting of the microglial response attenuated reactivity to LPS and, furthermore, altered behavior in mice, where exposed mice showed an inability to react appropriately to psychosocial stress (Lee et al., 2022). These impacts were mediated through the CB_1_R, as tested using receptor antagonists. These studies highlight the differential impact cannabis can have on the developing brain and the aged brain, where low doses may be detrimental to young animals, yet beneficial to aged animals.

The reduced production of pro-inflammatory cytokines with THC administration may occur through the inhibition of NF-κB signaling in microglia and astrocytes, as observed in cell culture experiments (Kozela et al., 2010; Rizzo et al., 2019). Interestingly, the overexpression of CB_1_Rs in adult mice undergoing experimental demyelination resulted in delayed onset and reduced severity of symptoms, whereas CB_1_R antagonism quickened symptom onset and increased the expression of inflammatory cytokines and NF-κB proteins (Lou et al., 2016, 2018). Furthermore, an alteration in cell state and expression profile (i.e., increase in pro-inflammatory cytokines and nitric oxide) was observed in cultured BV-2 cells treated with a CB_1_R antagonist (Lou et al., 2018).

It is clear that cannabinoids are involved in the microglial response to inflammation with respect to secretory profile and phagocytosis. This is the major way in which microglia may modulate myelination. However, it is important to note that there is still a paucity in *in vivo* experiments conducted with aged mice, emphasizing the need for increased research (Scipioni et al., 2022).

### 1.8. The impact of cannabis on astrocytes

The crosstalk that exists between microglia and astrocytes is essential for proper function and plasticity of the brain and maintenance of homeostasis (Jha et al., 2019; Matejuk and Ransohoff, 2020). However, in aging, certain aspects of this communication network become exacerbated. As discussed, the aged brain environment alters microglial state including their release of soluble factors. The concomitant impact aging has on astrocytes also induces a phenotypic switch that results in altered gene expression, perpetuation of inflammation, and recruitment of peripheral immune cells, which further exacerbates inflammation (Palmer and Ousman, 2018; Jha et al., 2019).

The ability for THC to inhibit NF-κB and reduce the release of IL-1β and TNF-α from microglia also extends to monocytes/macrophages and lymphocytes in human and rodent cell lines and primary cell cultures (Shivers et al., 1994; Kozela et al., 2010; Rizzo et al., 2019; Henriquez et al., 2020). This inhibition then translates into an observed reduction in the astrocytic release of IL-6 and monocyte chemoattractant protein-1 (MCP-1) in primary human cell cultures, thereby reducing inflammatory signaling and peripheral immune cell recruitment, respectively (Rizzo et al., 2019; Henriquez et al., 2020). Although IL-6 can be beneficial for many aspects of development including myelination, the chronically increased levels that are seen in aged humans and rodents are damaging and pro-inflammatory in nature (Ferrucci et al., 1999; Godbout and Johnson, 2004; Kimura and Kishimoto, 2010; Ritzel et al., 2016; Porcher et al., 2021).

This protective effect offered by THC is thought to be facilitated by the activation of CB_2_Rs, which is elevated in microglia and astrocytes within an inflammatory environment (Benito et al., 2008; Di Marzo et al., 2015; Cassano et al., 2017). However, other receptors may also play a role. Indeed, a recent study observed a marked inhibition of pro-inflammatory cytokines produced by IL-1β-stimulated primary cell cultured human astrocytes when pre−/co-treated with WIN55,212-2—a synthetic cannabinoid that displays similar effects to THC (Compton et al., 1992; Fields et al., 2022). This effect was independent of the CB_1_R and PPARs (Fields et al., 2022). Conversely, through PPARγ, CBD reduced pro-inflammatory cytokine release, inhibited NF-κB, and reduced GFAP expression in primary astrocyte cells stimulated with amyloid-β_1-42_ peptides, while also promoting neurogenesis in adult rats (Esposito et al., 2011). Furthermore, as discussed, CBD has the ability to diminish microglial cytokine production by scavenging ROS, diminishing NF-κB activity, reducing VCAM-1 levels and increasing the availability of endocannabinoid ligands. This may also lessen the extent to which astrocytes participate in peripheral immune cell recruitment and inflammation (Mecha et al., 2013). Indeed, a recent study found an association between CBD administration, reduced phosphorylation of NF-κB and reduced release of IL-6 from mouse primary cultured astrocytes stimulated with LPS (Wu et al., 2021).

However, cannabis includes both THC and CBD. Administration of Sativex^®^—an approved oromucosal spray (Health Canada and various European health agencies) containing THC (5 mg/kg) and CBD (5 mg/kg) for the treatment of symptoms associated with MS—reduced astrocyte reactivity and decreased the expression of pro-inflammatory cytokines released by microglia in a mouse model of MS (Feliú et al., 2015). Sativex^®^ also preserved myelin morphology in mice exposed to virus-induced demyelination.

Inflammation can also be induced by disrupting the proper communication between astrocytes and neurons. The deletion of CB_1_Rs from GABAergic neurons enhanced a phenotypic switch in astrocytes already associated with aging, including increased GFAP expression and amplified pro-inflammatory cytokine secretion in mice (Bilkei-Gorzo et al., 2018). Therefore, disruption of endocannabinoid signaling between neurons and astrocytes—which naturally occurs during aging—causes a deleterious transition in astrocytes that perpetuates cytokine-mediated damage. The application of cannabinoids may help since they directly act on astrocytes to diminish cytokine release (Sheng et al., 2005; Aguirre-Rueda et al., 2015; Rizzo et al., 2019; Fields et al., 2022).

Chronic inflammation was also identified as a major influencer of astrocytic gap junctions and hemichannels in rodents and humans (Bronzuoli et al., 2019; Peng et al., 2022). Typically, hemichannels remain mostly closed under “normal” conditions, but can be opened during pathological conditions; whereas the reverse is true for gap junctions (Peng et al., 2022). IL-1β and TNF-α released from microglia can open astrocytic hemichannels and reduce coupling between astrocytes in primary cell/slice cultures and *in vivo* in mice (Même et al., 2006; Retamal et al., 2007; Abudara et al., 2015; Vázquez et al., 2015b). The application of synthetic cannabinoids (e.g., WIN55,212-2) were able to reduce the microglial release of these factors and directly act on primary astrocyte cells from mice to reduce hemichannel activation, preventing astrocytic uncoupling and maintaining gap junctions (Froger et al., 2009; Gajardo-Gómez et al., 2017). Furthermore, direct activation of astrocytic CB_1_Rs was found to be required for the observed decrease in hemichannel activation, as determined by CB_1_R antagonism (Froger et al., 2009; Gajardo-Gómez et al., 2017). On the contrary, AEA (1 μM; topical application through a cortical cranial window) was shown to increase hemichannel activity in adult mice *in vivo*, resulting in a release of ATP that caused microglial process extension and migration toward the injury site (Vázquez et al., 2015b). Similarly, THC (5 mg/kg; i.p) has been shown to result in an increase in glutamate in the extracellular space by binding to astrocytic CB_1_Rs in mice, contributing to long term depression at synapses and impairments in working memory (Navarrete and Araque, 2010; Han et al., 2012).

During acute inflammatory conditions, this increase in hemichannel activity may be beneficial for mounting an immune response to an insult, however, the prolonged release of many factors (i.e., glutamate) from astrocytes in chronic conditions may be associated with altered synaptic plasticity and memory impairments (Navarrete and Araque, 2010; Han et al., 2012; Vázquez et al., 2015b; Labra et al., 2018). Alternatively, if cannabinoids are able to reduce the opening of hemichannels during a chronic inflammatory event, it may subsequently prevent excitotoxicity caused by excess glutamate and reduce the release of pro-inflammatory cytokines, thus maintaining neuronal and astrocyte viability (Froger et al., 2009; Gajardo-Gómez et al., 2017). Indeed, a recent review highlights a potential signaling cascade involving NF-κB, p38 and nitric oxide in which cannabinoids prevent the release of glutamate from hemichannels (Labra et al., 2018). It can also be hypothesized that the conserved function of gap junctions would facilitate proper communication between astrocytes and oligodendrocytes, promoting myelination (Papaneophytou et al., 2019).

In summary, elevated levels of inflammatory factors partly initiated by microglia and other recruited immune cells causes a phenotypic switch in astrocytes that contributes to functionally perpetuating inflammation *via* the release of pro-inflammatory cytokines and recruitment of peripheral immune cells. Cannabinoids can inhibit the release of these compounds (i.e., TNF-α, IL-6, MCP-1) by reducing the activity of pro-inflammatory pathways (e.g., NF-κB) in immune cells and astrocytes and increasing the availability of endocannabinoids. The subsequent decrease in pro-inflammatory factors and direct action of (endo)cannabinoids may also modulate hemichannel activity, thereby contributing to changes in extracellular homeostasis, synaptic activity and plasticity, as well as glial support functions. Although these results from studies using younger animals can provide information on how cannabinoids impact astrocytes and what this could mean for the process of myelination, the unique environment present in the aged brain makes extrapolation conjectural.

### 1.9. The impact of cannabis on oligodendrocytes and OPCs

As discussed, oligodendrocytes and OPCs are both essential for proper myelination and maintenance of myelin. Chronic increases in ROS, pro-inflammatory cytokines and other damaging compounds released from immune cells and astrocytes have the ability to damage mature oligodendrocytes and OPCs, resulting in myelination impairments (Pang et al., 2003; Jurewicz et al., 2005; French et al., 2009; Peferoen et al., 2014; Guttenplan et al., 2021; Sams, 2021). The ability for cannabinoids to reduce the release of pro-inflammatory cytokines from immune cells and astrocytes could therefore aid in the preservation of oligodendrocytes and OPCs. Furthermore, the antioxidant capacities offered by CBD could also protect these cells from oxidative stress.

However, in primary oligodendrocyte cell cultures from 12-day-old rats, CBD (100 nM–1 μM) resulted in mitochondrial dysfunction that led to increases in intracellular cytotoxic Ca^2+^ and ROS, which negatively impacted oligodendrocyte viability (Mato et al., 2010). Conversely, Mecha et al. (2012) found that CBD (1 μM) administered to inflammatory-induced primary oligodendrocyte cell cultures from the cortex of 2-day-old rats protected OPCs from oxidative stress and apoptosis. These studies found that these effects were independent of CB_1_R, CB_2_R, or PPARγ. The apparent discrepancy between studies is likely due to dosage and differences in age, region, and maturation of oligodendrocyte cells (Molina-Holgado et al., 2022).

Interestingly, CBD was also found to influence genes related to glycolysis—the major energy source for mature oligodendrocytes—and carbohydrate metabolism in oligodendrocytes (Rao et al., 2017; de Almeida et al., 2022). The data presented by de Almeida et al. (2022) suggests a slight downregulation of glycolysis in OPC and mature oligodendrocyte cell cultures (MO3.13). This finding is important because glycolysis produces lactate, an important energy metabolite transferred to neurons from oligodendrocytes, which has been shown to be essential for proper neuronal function in aged mice (12–24-months-old; Fünfschilling et al., 2012; Lee et al., 2012; Philips et al., 2021). Interestingly, the ablation of monocarboxylate transporter 1 (MCT1)—a lactate transporter—from OPCs resulted in hypomyelination and axonal degeneration in mature and older (18–24-months-old) mice, highlighting the understudied role played by OPCs in myelination among the adult brain (Philips et al., 2021). MCT1 expression naturally declines in the aging mouse brain, especially after 15-months of age (Ding et al., 2013; Philips et al., 2021). Therefore, further reductions in glycolysis due to CBD administration may prevent the beneficial support to neurons offered by oligodendrocytes and OPCs. Similarly, activation of mitochondrial CB_1_Rs with THC resulted in reduced glycolytic activity and lactate production in primary cultured astrocytes (0–1-days-old), which was hypothesized to contribute to the subsequent impaired neuronal function and altered behavior (e.g., social interaction deficit) in young adult (8–12-weeks-old) mice (Jimenez-Blasco et al., 2020). This reduction in astrocytic lactate would also likely have an impact on the oligodendrocyte lineage cells. For example, global inhibition of lactate production prevented remyelination in the corpus callosum after experimental demyelination in mice (~3-months-old), while OPC-rich primary cell cultures displayed heightened differentiation when lactate was added to the glucose medium (Ichihara et al., 2017). Perhaps this increase in OPC differentiation due to the presence of lactate can aid in remyelination by replacing the oligodendrocyte pool, which would warrant further investigation.

Although the literature is split with respect to OPC number in the aged brain, their ability to differentiate is severely impaired in humans and rodents (Sim et al., 2002; Yeung et al., 2014; Segel et al., 2019; Luan et al., 2021; Rivera et al., 2021; Zhang X. et al., 2021). Therefore, the encouragement of differentiation and protection of OPC viability offered by certain cannabinoids is of particular relevance (Ilyasov et al., 2018; Molina-Holgado et al., 2022). 2-AG was shown to promote the differentiation of OPCs into mature myelinating oligodendrocytes in primary mixed glial cell cultures from rats through the activation of CB_1_Rs and CB_2_Rs (Gomez et al., 2010). OPCs also have the ability to produce and release 2-AG themselves, indicating a potential autocrine and paracrine signaling mechanism that can further promote differentiation (Gomez et al., 2010, 2011). Pathways of note that have been shown to promote OPC differentiation through cannabinoid receptors are the phosphatidylinositol 3-kinase/Akt (PI3K/Akt) and mammalian target of rapamycin (mTOR), ERK/MAPK, and Rat sarcoma (Ras) homolog family member A/Rho-associated protein kinase (RhoA/ROCK; Molina-Holgado et al., 2002; Narayanan et al., 2009; Tyler et al., 2009; Gomez et al., 2010, 2011; Giacoppo et al., 2017; Sánchez-de la Torre et al., 2022; Wang et al., 2022).

These findings have prompted further research investigating the effect of THC on OPC differentiation. A recent study found that the application of THC (3 mg/kg; i.p) to young mice (6-days-old) and organotypic cerebellar cultures promoted the differentiation of OPCs (Huerga-Gómez et al., 2021). Furthermore, the same group found that THC (3 mg/kg; i.p) induced OPC differentiation and remyelination after experimentally-induced demyelination in the corpus callosum of adult (6–8-weeks-old) mice (Aguado et al., 2021). The modulation of OPC differentiation by THC is thought to be mediated by both CBRs, but mainly CB_1_Rs, since antagonism of CB_1_Rs prevented the beneficial effects observed. Furthermore, OPC(*Ng2*/Ai6)-CB_1_R-KO mice displayed impaired OPC differentiation and myelination in the corpus callosum throughout the examined lifespan (≤2-months-old; Sánchez-de la Torre et al., 2022). The impaired differentiation of OPCs lacking CB_1_Rs is thought to be partly due to an increase in RhoA/ROCK signaling. For example, THC (3 mg/kg, i.p) administered to WT mice resulted in reduced RhoA/ROCK proteins compared to vehicle exposed mice, leading to increased myelin-related proteins and enhanced OPC differentiation (Sánchez-de la Torre et al., 2022). However, OPC-CB_1_R-KO mice had increased levels of RhoA/ROCK proteins observed with a lower density of mature oligodendrocytes and reduced immunofluorescence against myelin-related proteins, which did not significantly change with THC administration (Sánchez-de la Torre et al., 2022). These findings highlight the importance of the CB_1_R in OPC differentiation, and its relationship with the RhoA/ROCK pathway. Furthermore, these findings are supported by previous studies that have shown the importance of RhoA/ROCK signaling in OPC differentiation (Baer et al., 2009; Pedraza et al., 2014). Similarly, the application of WIN55,212-2 (i.p) resulted in improved remyelination after experimentally-induced demyelination in mice (6–7-weeks-old) when administered at a dose of 0.5 mg/kg; whereas a dose of 1 mg/kg impaired remyelination (Tomas-Roig et al., 2020). The negative effects produced by 1 mg/kg of WIN55,212-2 are hypothesized to be due to stronger reductions in Ca^2+^ influx, resulting in reduced neuronal activity, which possibly hinders activity-dependent myelination (Tomas-Roig et al., 2020). Overall, it is evident that at the right dose cannabinoids can alter myelination and promote the maturation of oligodendrocyte lineage cells.

In summary, cannabinoids at the right doses have the ability to aid in remyelination and promote OPC differentiation after pathological insults. As outlined throughout this review, cannabinoids act on a plethora of different cell types and different targets within those cells, which directly and indirectly impact myelination. Although cannabinoid-induced alterations to microglia and astrocytes can influence OPC differentiation and oligodendrocyte function indirectly, it is also important to keep in mind that cannabinoids directly act on OPCs to influence maturation and function. However, there are a lack of studies regarding the healthy aged brain. Although the aged brain does have increases in ROS, pro-inflammatory cytokines, and a general reduction in glial cell function, the extent of these changes and the dynamics at play are inherently different than those observed after the application of LPS or experimentally-induced demyelination at younger ages. Furthermore, findings from cell line and primary cell cultures need to be validated in *in vivo* experiments. Therefore, although studies outlined here point toward a role played by cannabinoids in myelination, more studies specifically looking at their impact in the healthy aged brain are required to confirm if these findings do indeed translate. Nonetheless, the evidence provided thus far suggests that cannabinoids may help promote myelination in the aged brain. Neuroimaging studies offer a different approach to visualize how cannabinoids impact brain communication and myelination, which allows elucidating large-scale changes that may result from the impact they have on glial cells and neuronal function.

### 1.10. The impact of cannabis on the human brain

Neuroimaging studies are valuable non-invasive techniques used to visualize large-scale changes in activity, connectivity, and structural alterations in the brain. DTI is a magnetic resonance imaging (MRI) technique that differentiates the degree and direction of water diffusion within an allotted space (Pierpaoli and Basser, 1996). Three main parameters are given by DTI for white matter: fractional anisotropy (FA), mean diffusivity (MD) and radial diffusivity (RD; Alexander et al., 2007). Generally, as described by Becker et al. (2015), increases in FA and decreases in MD and RD reflect increases in myelination.

The DTI literature encompassing cannabis use and myelin integrity is not consistent. Difficulties with respect to accurate reporting on usage and concentration of cannabis, and the ratio of cannabinoids—not to mention other confounding factors such as lifestyle and metabolism—make human cannabis studies increasingly difficult to perform. Furthermore, the DTI metrics that are used as proxies of myelin integrity do not specifically identify myelin abnormalities, but detect differences in water diffusion—a parameter that can be altered by axon packing density, axon caliber, and more confounding factors (Chang et al., 2017). However, it has been shown that FA can correlate with myelination quite accurately (Chang et al., 2017). With this in mind, some tentative directions can be ascertained.

Studies focusing on adolescent and young adults who heavily use cannabis have drawn cautious conclusions. Overall, reductions in FA and increases in RD and MD have been observed with chronic exposure to cannabis (Arnone et al., 2008; Ashtari et al., 2009; Gruber et al., 2014; Becker et al., 2015; Shollenbarger et al., 2015). However, Cousijn et al. (2022) did not find any significant differences between chronic, sporadic or control groups, while noting an association between reduced FA and the onset of regular cannabis use at younger ages (<18-years-old). This finding is generally supported in the literature, with many studies indicating that an earlier age of onset correlates with reduced myelin integrity and cognition (Gruber et al., 2012, 2014; Lisdahl et al., 2013; Orr et al., 2016).

Other studies with larger age ranges (~22–55-years of age) revealed similar results with respect to heavy cannabis use. Jakabek et al. (2016) found reductions in FA in the forceps minor across all age ranges (18–55-years of age), but RD differed depending on age, with younger users having reduced RD and older users having higher RD. Similarly, Manza et al. (2020) reported compromised myelin integrity in heavy cannabis users compared to controls within the same age range. However, Filbey et al. (2014) observed an increase in FA and a decrease in RD, suggesting improvements in white matter integrity, although protracted use did result in a reversal of these findings.

On the contrary, a recent DTI study found that adults (47.85 ± 17.42-years of age) who use medical cannabis with moderate levels of CBD and low levels of THC have significantly increased FA and reduced MD in multiple white matter regions—notably the genu of the corpus callosum—after 6 months of use (Dahlgren et al., 2022). An important distinction between this study and the previously mentioned studies is that the participants were using cannabis for medicinal purposes, not recreationally. As stated by Dahlgren et al. (2022), the medicinal use of cannabis likely involves drastically different characteristics (i.e., age of onset, ratio of cannabinoids), potentially resulting in the increases in myelin integrity found in this study. Furthermore, the medicinal use of cannabis is likely to help treat symptoms associated with increased levels of inflammation, perhaps altering cannabinoid function compared to recreational users. These findings are supported by the numerous animal studies highlighted in this review that show that lower doses of cannabinoids administered less frequently are more beneficial than the reverse.

Despite indications that the brains response to cannabis substantially changes with age, to the best of our knowledge, no DTI study looking at the integrity of myelin has yet been performed in cannabis using seniors, creating a substantial gap in the literature. Through the use of functional MRI, functional connectivity—the statistical relationship that exists between different regions of the brain that are necessary for cognitive processes—can be quantified (Gaudet et al., 2020). Importantly, these functional connections are supported by, and significantly associated with white matter pathways (Hunt et al., 2016; Meier et al., 2016; Huntenburg et al., 2017; Vandewouw et al., 2021). Again, few studies are available for adults over the age of 60. However, one study found increased functional connectivity between the anterior cerebellum and the hippocampus, and with the posterior parahippocampal cortex in cannabis users aged 60–80-years-old (Watson et al., 2022). The authors suggest this finding may indicate a potential benefit of cannabis in the aged brain, although the extent and appearance of these potential benefits are still unclear.

Due to the paucity of studies conducted in older human adults, it is difficult to identify the impact cannabis has on myelination in this population using neuroimaging techniques at this time. Regardless, this evidence forms a basis for future exploration.

## 2. Conclusion

The studies examined in this review highlight the potential for cannabinoids to aid in myelination in the aged brain. At certain doses, cannabinoids have the ability to reduce the release of pro-inflammatory cytokines from microglia and astrocytes, scavenge ROS, and promote OPC differentiation in a cell-autonomous manner, resulting in improved myelination after an inflammatory stimulus or demyelination. Furthermore, there is evidence suggesting that some cannabis use in the adult and older population may improve white matter integrity. However, the extreme lack of studies on this topic in the healthy aged brain currently prevents any definitive conclusions from being drawn. Therefore, future studies looking at the impact cannabinoids have on myelination in the aged brain and how this alters behavior and cognition should be performed. As a note, the myelination process in the peripheral nervous system is vastly different than in the CNS and was beyond the scope of this review, but cannabinoids may also influence this process. These studies will not only shed light onto how cannabinoids impact myelination, but will also add vital information as to how the endocannabinoid system contributes to modulating cognition in the aged brain.

## Author contributions

CM: conceptualization, writing—original draft preparation, writing—review and editing, and visualizations. HV: conceptualization and writing—review and editing. M-ÈT: conceptualization, writing—review and editing, supervision, and funding acquisition. All authors contributed to the article and approved the submitted version.

## Funding

This work was supported by research grants from the Canadian Institutes of Health Research (CIHR) and Natural Sciences and Engineering Research Council of Canada (NSERC) awarded to M-ÈT. HV is the recipient of a CIHR postdoctoral fellowship and is a Michael Smith Health Research BC Research Trainee.

## Conflict of interest

The authors declare that the research was conducted in the absence of any commercial or financial relationships that could be construed as a potential conflict of interest.

## Publisher’s note

All claims expressed in this article are solely those of the authors and do not necessarily represent those of their affiliated organizations, or those of the publisher, the editors and the reviewers. Any product that may be evaluated in this article, or claim that may be made by its manufacturer, is not guaranteed or endorsed by the publisher.
